# Community severance and mental health-related hospital visits in New York City

**DOI:** 10.1097/EE9.0000000000000482

**Published:** 2026-04-27

**Authors:** Jaime Benavides, Gali Cohen, Jeff Goldsmith, Marianthi-Anna Kioumourtzoglou

**Affiliations:** a Department of Epidemiology, School of Public Health, Brown University, Providence, Rhode Island; b Department of Environmental Health Sciences, Columbia University Mailman School of Public Health, New York City, New York; c Department of Epidemiology and Preventive Medicine, School of Public Health, Gray Faculty of Medical & Health Sciences, Tel Aviv University, Israel; d Department of Biostatistics, Columbia University Mailman School of Public Health, New York City, New York; e Institute at Brown University for the Environment and Society, Providence, Rhode Island

## Abstract

**Background:**

Mental health disorders are more prevalent in urban areas; urban living has been linked to increased risk of anxiety, mood, and schizophrenia disorders. These associations may be partially explained by the social and physical urban environment. While traffic-related exposures such as air pollution and noise have been linked to adverse mental health outcomes, the role of road infrastructure and traffic in severing communities and breaking down the social fabric—termed community severance—remains understudied.

**Methods:**

We conducted ZIP code-level analyses (2011–2014) to investigate the association between mental health hospital visits and community severance in New York City (NYC), using annual counts of mood-, anxiety-, adjustment-, and schizophrenia-related hospital visits from the New York State Department of Health Statewide Planning and Research Cooperative System database. Community severance was quantified using the community severance index (CSI). We employed generalized additive mixed models to characterize potentially nonlinear associations, adjusting for potential confounders. In secondary analyses, we further adjusted for black carbon to obtain traffic pollution-independent CSI estimates and evaluated potential effect modification by age through stratified analyses.

**Results:**

Each interquartile range increase in CSI was associated with higher rates of schizophrenia-related hospital visits (rate ratio [RR] = 1.13, 95% confidence interval [CI] = 1.00, 1.27). Associations for mood (RR = 1.07; 95% CI = 0.98, 1.16), anxiety (RR = 1.06; 95% CI = 0.99, 1.14), and adjustment disorders (RR = 1.07; 95% CI = 0.97, 1.19) were also positive. Results were similar after black carbon adjustment. We found no clear evidence of effect modification by age.

**Conclusion:**

Our findings indicate an association between higher levels of community severance and schizophrenia-related hospital visits in urban areas, independent of traffic-related air pollution.

**What this study adds**

This study adds new insight into how the urban environment can impact mental health, beyond well-known environmental risk factors like air pollution. By using an index for community severance, the physical and social disconnection caused by road infrastructure and traffic, we examined whether neighborhoods with higher levels of community severance experience different mental health outcomes. We found that higher levels of community severance are associated with increased schizophrenia-related hospital visits. These findings draw attention to an overlooked urban exposure and highlight the need for further research on how features of city design may influence mental health.

## Introduction

Mental health disorders are a rising global concern, accounting for 16% of the disability-adjusted life years in 2019 with an associated economic value of 5 trillion USD.^1^ Population aging and urbanization are two converging demographic trends, meaning that an increasingly large and older segment of the population will be aging within urban environments in the coming decades. Growing urbanization poses substantial challenges for mental health, as mental health issues accumulate in cities compared with rural settings.^2^ For instance, urban living reportedly has substantial influence on elevated prevalence of anxiety (+21%), mood disorders (+39%), and about double the risk of schizophrenia diagnoses.^3,4^ This link can be potentially explained by the impact of social and physical urban environments.^5^ Most of the US population (82%) lives in urban areas, a proportion projected to rise to 89% by 2050.^6^ Identifying urban modifiable risk factors of mental health disorders, therefore, is of critical importance.^7^ To enable urban designs that mitigate societal mental health risks and promote mental well-being, we need to understand the extent to which the characteristics of urban environments influence mental health.

Different features of the built environment have been associated either with being detrimental to or supportive of mental health.^5^ For instance, in recent years, multiple studies have consistently linked air pollution to poor mental health^8–11^ and acute psychiatric episodes.^12,13^ In contrast, access to green and blue spaces has been associated with lower symptoms of depression,^9,14^ mental distress,^15,16^ and schizophrenia risk, especially when these exposures were experienced in child-hood.^17–19^ Social factors like low social cohesion and high crime also increase psychosis risk in children.^20^ The design and use of public spaces in cities, such as prioritizing roads and traffic versus communal or green areas, can either alleviate or worsen mental health outcomes.^21^

Traffic-related exposures (e.g., air pollution and noise) have a detrimental impact on human health,^22–24^ especially in urban areas, where most of the population and road traffic concentrate. However, the health implications of the physical separation imposed by road infrastructure and motorized road traffic, named community severance, have been understudied. Community severance limits access to goods, services, or social connections, breaking down the social fabric. Community severance may impact mental health through different pathways, including discouraging walking and physical activity, increasing psychological stress due to road safety concerns,^25,26^ and limiting social contacts among community residents.^27^ Social connectedness is a key factor in both preventing depressive symptoms and alleviating mood disorders.^28–30^ In contrast, isolation tends to co-occur with loneliness,^31^ which may in turn directly heighten anxiety and depression^32^ and intensify psychotic symptoms.^33,34^ Despite strong mechanistic plausibility, the association between community severance and mental health has not yet been investigated.

In this work, we assessed the association between community severance and mental health-related hospital visits as measured by hospital visits due to anxiety, mood, adjustment, and schizophrenia disorders. We recently developed the first community severance index (CSI) in New York City (NYC), applying a pattern recognition algorithm adapted from computer vision.^35^ We leveraged the New York (NY) Department of Health Statewide Planning and Research Cooperative System (SPARCS) database to obtain all emergency department visits and hospital admissions in NYC (2011–2014). To the best of our knowledge, this is the first-ever study examining the impact of community severance on mental health, leveraging the rich data resources of the largest city in the United States.

## Methods

### Study population

We acquired hospital records from 2011 to 2014 from the NY Department of Health’s SPARCS database. SPARCS gathers administrative data from all nonmilitary acute care facilities in NY, encompassing approximately 98% of all hospital visits in the state. As of 2015, SPARCS included data from 222 acute care facilities.^36^ Our analysis included both inpatient and outpatient visits for all ages (the term “hospital visits” is used throughout to refer to both for simplicity). Each hospital visit record has information on International Classification of Diseases, Ninth Revision, Clinical Modification (ICD-9-CM) diagnosis codes, along with patient residential ZIP Code, date of hospital visit, age, sex, and visit type (inpatient vs. outpatient). We included hospital visits from each of the five NYC boroughs (Bronx, Brooklyn, Manhattan, Queens, and Staten Island). We excluded ZIP codes with no inhabitants (i.e., population of zero or one) and ZIP codes in which the JFK airport is located. This study received approval from the Institutional Review Board at the Columbia Mailman School of Public Health and was classified as exempt from requiring informed consent. Area-level exposures and covariates were calculated at the ZIP Code Tabulation Area level, which provides a consistent geographic approximation of ZIP Codes in NYC. For analytical purposes, ZIP Code Tabulation Areas were used as the spatial unit and are referred to as “ZIP codes” throughout the manuscript for simplicity.

### Mental health outcomes

We leveraged data for the most common causes for mental health-related hospital visits in NYS: anxiety, mood, adjustment, and schizophrenia and other psychotic disorders (referred to as schizophrenia disorders from this point forward). Classifications were determined in a previous work by our group^37^ using the Clinical Classifications Software algorithm,^38^ which is frequently employed in epidemiologic studies to organize ICD codes into clinically meaningful categories (Supplementary Table 1, https://links.lww.com/EE/A424). For each cause, we considered a hospital visit as a case if it contained at least one matching code within the first four ICD-9-CM code categories. This means that a single hospital visit could be attributed to multiple causes. For all our main analyses we focused on 2011–2014. We spatio-temporally aggregated mental health hospital visits to annual counts per ZIP code. Data on hospital visits were provided in aggregated form and did not include unique patient identifiers; therefore, counts reflect hospital visit events rather than unique individuals.

### Community severance index

Our team has developed a standardized, city-wide, highly spatially resolved index of community severance.^35^ In our previous NYC study, we used geolocated data and a computer vision and signal processing algorithm for dimensionality reduction and pattern recognition named Principal Component Pursuit (PCP).^39,40^ Briefly, we combined PCP with factor analysis to estimate the CSI. The normalized scores of the CSI range from 0 to 1, with higher values indicating greater community severance. We used public data for 2019 at the census block group level on road infrastructure, road traffic activity, and pedestrian infrastructure. Using factor analysis, we identified one factor related to community severance explaining 74% of the variation in the PCP low-rank matrix. This factor is characterized by positive loadings of road infrastructure and traffic activity and negative loadings of pedestrian infrastructure.^35^

Using the same conceptual and analytical framework as for the 2019 CSI,^35^ we estimated CSI for the 2011–2014 period. We relied on the same data categories and, whenever possible, the same data sources as in the original construction, selecting earlier versions (e.g., 2012–2014 releases) to align temporally with the 2011–2014 health data. Details on the inputs and the estimation of the 2011–2014 CSI can be found in the Supplement (Section S1, https://links.lww.com/EE/A424).

Since the mental health data are available at the ZIP code level, we aggregated CSI to ZIP codes using population weights derived from 2010 Decennial Census block group total population, so that areas with larger populations contributed proportionally more to the corresponding ZIP code-level aggregated CSI values.

### Covariates

We obtained ZIP code-level socioeconomic status (SES) data from the 5-year averages of American Community Survey^41^ that may affect mental health outcomes and may also be correlated with CSI, that is, percentage of residents below the poverty threshold and racial and ethnic distributions (Black and Hispanic percentages). We also obtained population density from American Community Survey as inhabitants*/*km^2^. We used data from the Protected Areas of the United States that are Accessible and Recreational dataset to assess green space access, which measures accessible and recreational parks in the contiguous United States.^42^ This dataset is based on the nation’s inventory of protected areas, restricted to those areas accessible and recreational for the public, and it was parameterized as the percent of ZIP Code designated as accessible recreational green or blue space, including water bodies that support outdoor recreation. These covariates were assessed as potential confounders using directed acyclic graphs (DAGs; Figure S1, https://links.lww.com/EE/A424) and expert knowledge.

Additionally, we obtained annual predicted black carbon (BC) concentrations—a pollutant commonly used as a surrogate for traffic emissions-related air pollution—from the New York City Community Air Survey (NYCCAS) for the period 2011–2014.^43^ New York City Community Air Survey provides annual surfaces of BC at 300 m^2^ grid resolution; we aggregated the gridded BC predicted concentrations to ZIP Code-level averages, using population weights, similar to the aggregation performed for CSI. In our analysis, BC was considered a potential mediator rather than a confounder, as community severance captures traffic-related features that also influence local air pollution and, in turn, may affect mental health. Adjusting for BC would therefore block this indirect pathway, yielding CSI estimates independent of traffic-related air pollution.

### Statistical analysis

We ran quasi-Poisson generalized additive mixed models (GAMMs), with ZIP code-level population as the offset, to estimate the potentially nonlinear associations between CSI and annual mental health hospital visits at the ZIP code level adjusting for SES, population density, and green space access. For this analysis we assumed that CSI did not meaningfully change over the study period (2011–2014) and was thus included as a nontime-varying exposure in the models. In our main analysis, we trimmed CSI values larger and lower than 2 standard deviations from the mean to attenuate the influence of outliers (i.e., 24 values, 3.4%). We additionally adjusted for borough to account for potential confounding factors varying by borough and included a random intercept for ZIP Code to capture unobserved, time-invariant differences and within–ZIP code clustering across the study period. Initially, we included penalized splines to flexibly characterize the associations between CSI and mental health hospital visits. We interpreted estimated degrees of freedom (edf) equal to 1 as indicative of a linear association. In those cases where edf *>*2, we considered the association nonlinear. For edf ∈ [1, 2], we visually inspected the estimated curve to qualitatively assess its shape. If relationships were nonlinear, CSI was modeled using a natural spline with 4 df, which was preferred over penalized splines in the final models due to the latter’s greater flexibility and sensitivity to influential observations. We present results from linear models as rate ratios (RR) and 95% confidence intervals (CI) for each interquartile range (IQR) increase in CSI. For nonlinear results, we present the curve of the association across the CSI range and set the 25th percentile of CSI as the reference for plotting the results.

### Sensitivity analysis

As a sensitivity analysis, we estimated the association between CSI and cause-specific mental health hospital visits, including the CSI potential outliers (i.e., values larger and lower than 2 standard deviations from the mean CSI).

### Secondary analyses

The associations between CSI and cause-specific mental health hospital visits may be mediated by traffic-related air pollution, which has been identified as a risk factor for mental health outcomes in previous studies.^44^ As a secondary analysis, therefore, we included annual average concentrations of BC as a covariate in the models. This analysis was not meant to estimate the potential mediating role of BC on the CSI-mental health association. Rather, we aimed to estimate the independent effect of CSI on cause-specific mental health hospital visits excluding the potential path through traffic-related air pollution.

To account for potential confounding due to temporal trends on the BC–cause-specific mental health hospital visits, we included calendar year as a categorical variable in all secondary analyses models. In addition, adjustment for calendar year allowed us to block the collider path that was opened once we included BC in the models (Supplemental Figure 1, https://links.lww.com/EE/A424).

Older adults may be more severely impacted by limited access to goods and social interactions in highly severed communities. As another secondary analysis, thus, we evaluated potential effect modification by age by stratifying models into two groups: patients younger than 65 years and patients aged 65 years and older.

## Results

**Figure 1.**
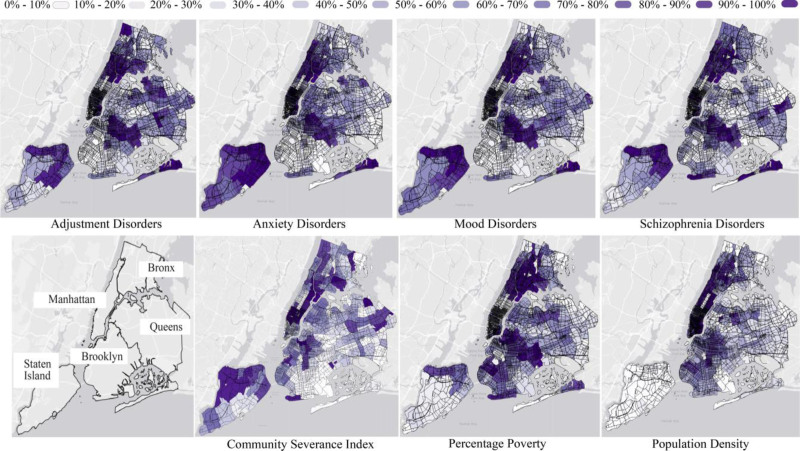
Spatial distribution of ZIP code-level hospital visit rates (visits per 10,000 inhabitants) for adjustment, anxiety, mood and schizophrenia disorders (top panel), along with the boroughs, community severance index, poverty rate, and population density (bottom panel). Values of these variables are presented in deciles. See Figure S4, https://links.lww.com/EE/A424 for the spatial distribution of the other covariates.

We evaluated the association between CSI and cause-specific mental health hospital visits in NYC for the period from 2011 to 2014. Table 1 presents the annual distributional characteristics of mental health-related hospital visits across the 176 NYC ZIP codes per 10,000 inhabitants. For most outcomes, we observed higher hospital visit rates in Staten Island and the Bronx. In general, the rate in Brooklyn was slightly lower than the rates at the other boroughs. For instance, ZIP codes in Brooklyn registered an annual median of 329 mood and 143 anxiety disorder hospital visits per 10,000 inhabitants per year, respectively. In contrast, for the same period, the medians of mood and anxiety disorder hospital visits per 10,000 in NYC overall were 404 and 168, respectively. The spatial distribution of both mood and schizophrenia disorder hospital visits during the period 2011–2014 showed similar spatial patterns as poverty rate across NYC ZIP codes, with increasing rates in the Bronx and the northern sides of Brooklyn and Staten Island (Figure 1, top row).

**Table 1.** Distributional characteristics (median [interquartile range, IQR]) of annual cause-specific mental health-related hospital visit rates across New York City ZIP codes (N = 176) during 2011–2014, presented as visits per 10,000 inhabitants per year

Borough	Mood disorders	Anxiety disorders	Adjustment disorders	Schizophrenia and other
	(N = 1,572,058)	(N = 625,894)	(N = 120,872)	Psychotic disorders (N = 46,962)
NYC-wide	404 (282, 593)	168 (118, 222)	29 (18, 46)	10 (6, 18)
Brooklyn	329 (168, 620)	143 (99, 210)	25 (15, 44)	12 (7, 18)
Manhattan	368 (245, 672)	181 (123, 282)	27 (15, 49)	11 (5, 22)
Queens	398 (313, 459)	152 (115, 186)	29 (18, 40)	7 (5, 11)
Staten Island	615 (459, 828)	278 (218, 337)	39 (25, 54)	12 (6, 21)
The Bronx	519 (316, 695)	176 (136, 226)	37 (24, 53)	16 (10–20)

Using data representative of the 2011–2014 period, we obtained two latent factors, consistent with the 2019 CSI. The first factor explained approximately 75% of the shared variability in the PCP low-rank matrix and exhibited strong positive loadings for road infrastructure and traffic-related measures, and negative loadings for pedestrian-oriented infrastructure (Figure S2, https://links.lww.com/EE/A424). This factor closely aligned with the conceptual definition of community severance and was retained as the CSI for 2011–2014. All subsequent analyses were conducted using these CSI scores ranging from −1.9 to 1.9.

CSI also differed by borough (Figure 1 and Table 2), with higher levels in Manhattan and lower levels in Queens. A total of 3.4% of the CSI values were smaller or larger than mean ±2 × its standard deviation and, thus, classified as potential outliers.

**Table 2.** Distributional characteristics, presented as median (interquartile range, IQR), of exposure (CSI) and covariates across NYC ZIP codes (N = 176)

Variable	NYC-wide	Brooklyn	Manhattan	Queens	Staten Island	The Bronx
CSI	0 (−0.3, 0.4)	−0.1 (−0.5, 0.2)	0.3 (−0.2, 0.8)	−0.1 (−0.5, 0.1)	0.2 (−0.1, 0.4)	0.1 (0, 0.4)
Pop. Dens.^a^	14 (8, 24)	16 (12, 21)	31 (22, 39)	9 (6, 14)	3 (3, 5)	17 (9, 26)
Poverty (%)	15.4 (9.4, 24)	21.9 (15, 31.5)	11.1 (7.5, 24.2)	13 (8.8, 17.6)	10.2 (7.2, 20.4)	32.4 (15.7, 39.8)
Black (%)	7.9 (2.8, 32.7)	20 (4.8, 58.4)	6 (2.8, 12.1)	6.5 (2.3, 18.7)	4.4 (2, 26.2)	32 (22.7, 38.1)
Hispanic (%)	18.2 (10.7, 36.3)	14.4 (10.4, 21.9)	13.1 (7.4, 22.6)	18.4 (12.9, 31)	13.7 (10.9, 27.9)	59 (36.1, 68.2)
Green spaces (%)	6.1 (2.2, 16.7)	3.9 (2.7, 11.3)	6.1 (2, 20.2)	4.1 (2, 15.8)	17.7 (6.4, 23.7)	8.7 (4.3, 16.7)
Black Carbon^b^	1.1 (0.9, 1.3)	0.9 (0.8, 1.1)	1.4 (1.3, 1.6)	0.9 (0.8, 1)	0.8 (0.7, 0.8)	1.2 (1.1, 1.3)

CSI and all other variables were assessed for the years of the study (2011–2014).

a Population density presented as 1,000 inhabitants*/*km^2^.

b Black Carbon concentration: µg/m^3^.

CSI indicates Community Severance Index; Pop. Dens., population density.

Manhattan had double the population density compared with the NYC-wide median. In contrast, the population density in Staten Island was lower compared with the other NYC boroughs, with higher levels of green spaces. BC annual mean levels were higher in Manhattan (median = 1.4; IQR = 1.3–1.6 µg/m^3^) and the Bronx (median = 1.2; IQR = 1.1–1.3 µg/m^3^) compared with the NYC-wide average (median = 1.1; IQR: 0.9–1.3 µg/m^3^).

CSI, BC, and population density were positively correlated (correlations ranged between 0.4 and 0.7; Figure S3, https://links.lww.com/EE/A424). The correlations between these three variables and green spaces ranged between 0.0 and −0.3.

### Association between community severance index and mental health hospital visits

**Figure 2.**
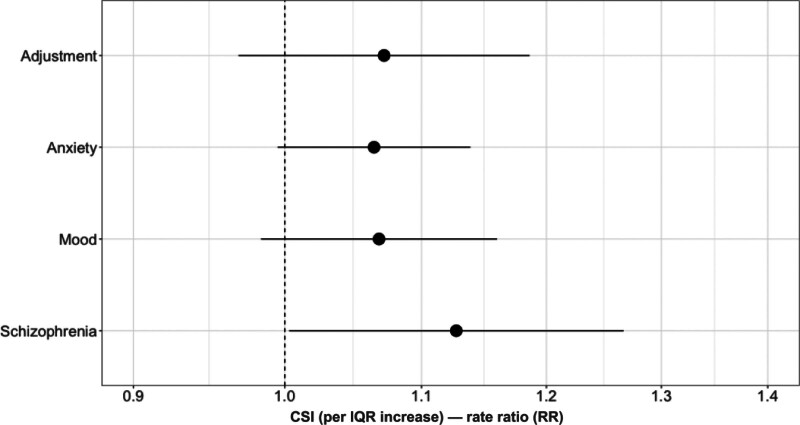
Associations between ZIP code-level community severance index (CSI) and cause-specific mental health hospital visits. The vertical dotted line represents the null; horizontal lines are the 95% confidence intervals for each estimated rate ratio (RR) per each interquartile range (IQR) increase in CSI, plotted on a logarithmic scale. Models were adjusted for population density, Black (%), Hispanic (%), poverty rate, borough, and green spaces. See Supplemental Table S2, https://links.lww.com/EE/A424 for corresponding numeric data.

In the main analysis, we did not detect any deviations from linearity in the relationships between CSI and all causes of mental health hospital visits (Figure 2 and Table S2, https://links.lww.com/EE/A424). We estimated that for each IQR increase in CSI, the rate for mood disorder-related visits was 1.07 times higher (RR = 1.07, 95% CI = 0.98, 1.16), for anxiety RR = 1.06 (95% CI = 0.99, 1.14), and for adjustment disorders RR = 1.07 (95% CI = 0.97, 1.19). Finally, each IQR increase in CSI levels per ZIP code was associated with a 13%-increase in annual hospital visits for schizophrenia disorder (RR = 1.13, 95% CI = 1.00, 1.27).

### Sensitivity analysis

When including potential CSI outliers, we found consistent results compared with the main analysis for adjustment and schizophrenia disorders (Figure S5, https://links.lww.com/EE/A424). An IQR increase in CSI was associated with slightly higher annual rates of anxiety-related hospital visits (RR = 1.08, 95% CI = 1.02, 1.15). Also, for mood disorders we observed a nonlinear relationship (Figure S6, https://links.lww.com/EE/A424). Rate ratios for mood disorder-related hospital visits increased with higher levels of CSI, showing an overall monotonic upward trend. The association appeared steeper at both lower and higher CSI values, indicating stronger increases in risk at the extremes of the CSI distribution.

### Secondary analyses

When we additionally included BC concentrations in the model to block the potential pathway through traffic-related air pollution, the estimated associations were slightly attenuated, with CIs crossing the null, but were generally similar to those observed in the main analysis (Figure S7, https://links.lww.com/EE/A424). For example, for CSI and schizophrenia-related hospital visits, the RR was 1.11 (95% CI = 0.98, 1.26, Table S4, https://links.lww.com/EE/A424).

In age-stratified analyses, we did not observe clear evidence of effect modification by age. Among individuals aged 65 years and older, we observed some deviations from linearity in the associations between CSI and hospital visits for anxiety, mood, and adjustment disorders (Figure S8, https://links.lww.com/EE/A424). For anxiety and adjustment disorders, higher CSI values were associated with higher hospital visit rates at the upper end of the exposure distribution, although estimates in this age group were less precise. For schizophrenia, the positive association observed in the main analysis was similar among individuals younger than 65 years and those aged 65 years and older (Figure S8, https://links.lww.com/EE/A424).

## Discussion

To the best of our knowledge, this is the first study to examine the associations between community severance, as measured by CSI, and cause-specific hospital visits for mental health disorders, that is, for anxiety, mood, adjustment, and schizophrenia disorders. By analyzing data across multiple neighborhoods in NYC, we found that higher levels of community severance, characterized by dense road infrastructure, high-traffic volume, and limited pedestrian infrastructure, were associated with higher rates of schizophrenia-related hospital visits. We also observed positive associations for mood, anxiety, and adjustment disorders, although their CIs included the null.

Although schizophrenia and related disorders are primarily driven by genetic liability, environmental exposures are estimated to account for 15–40% of overall risk.^45^ A substantial body of research shows that urban environments are associated with elevated risks of anxiety, mood, and psychotic disorders, and city living is one of the most consistently observed area-level predictors of mental health outcomes.^3,5,46–48^ Socio-environmental stressors such as crime, socioeconomic deprivation,^49^ low educational attainment,^50^ and adverse physical conditions may interact with genetic vulnerabilities to shape mental health outcomes.^51^ However, the specific features of urban environments that contribute most strongly to these risks remain incompletely understood. Much of the existing literature has focused on environmental exposures such as air pollution or limited access to green space.^8–10,52–55^ Our study adds to this scientific literature by using a derived index to systematically quantify community severance, an understudied aspect of the built environment. Our findings identify community severance as a potentially relevant urban characteristic associated with schizophrenia-related hospital visits, with suggestive signals also observed for mood and anxiety disorders. Further studies in other geographic settings will be important to determine whether similar associations are observed beyond NYC.

The observed association between community severance and schizophrenia-related hospital visits may be explained by interconnected socio-environmental mechanisms. Although schizophrenia was the least common outcome in our dataset, and thus subject to limited statistical power, we observed the strongest associations for this disorder. This pattern aligns with broader evidence that urbanicity may have the most pronounced effects on nonaffective psychosis, with weaker or more variable associations for mood and anxiety disorders.^5^ The generally positive associations we observed for anxiety and mood disorders, although crossing the null, together with the stronger association for schizophrenia are consistent with our initial hypothesis and the possibility that highly severed neighborhoods concentrate environmental stressors that may adversely affect mental health.

Our findings also align with research by Pun et al.,^56^ who demonstrated a link between residential proximity to roads, a component of the CSI, and increased depression and anxiety in older adults, potentially mediated by factors such as loneliness and air pollution. Community severance not only restricts physical mobility but also limits opportunities for social connection and access to restorative environments such as green spaces.^57^ It may also operate through additional pathways, including poorer road safety, increased exposure to traffic-related stressors, and higher accident risk. Severance contributes to the accumulation of environmental stressors by replacing open spaces—where children could safely play, residents could connect socially,^58^ and communities could foster cohesion—with extensive road networks dominated by vehicular traffic.^25^ Seminal work in San Francisco by Appleyard and Lintell,^27^ later replicated in the United Kingdom,^59^ demonstrated that residents in high-traffic areas reported fewer social interactions and weaker neighborhood cohesion. These environments may also contribute to increased family stress and social isolation, both of which are associated with greater psychiatric vulnerability. These psychosocial disruptions may be amplified in contexts where other environmental stressors are prevalent, creating cumulative burdens and reducing opportunities for stress relief.^5,60^ In addition, traffic-related environments increase the risk of road injuries, and survivors of traffic accidents may experience psychiatric morbidity lasting up to 24 months, with prevalence rates approaching 50%.^61^

Beyond these social and safety-related pathways, severed neighborhoods may also face disproportionate exposure to climate-related hazards. Extreme weather events, such as heatwaves and flooding, are strongly associated with adverse physical and mental health outcomes.^62–64^ Community severance can exacerbate these risks by fostering loneliness—a well-established determinant of poor health—and reducing the likelihood that residents check on vulnerable neighbors during crises. Severed neighborhoods are also more likely to be inhabited by marginalized populations with limited adaptive resources, further compounding the health and social consequences of climate hazards.^64,65^

These social-environmental disruptions provide a pathway through which biological mechanisms of stress and inflammation may be activated. Chronic exposure to psychosocial stressors in severed communities may disrupt the hypothalamic-pituitary-adrenal axis regulation, leading to cognitive dysfunction and long-term neuronal changes.^66^ Hypothalamic-pituitary-adrenal axis dysregulation is increasingly recognized as a schizophrenia risk factor.^67^ Stress pathways implicated in schizophrenia also overlap with those in depression,^67,68^ complicating diagnostic differentiation but underscoring shared vulnerability to socially fragmented environments. Taken together, these findings suggest that community severance could increase schizophrenia risk by intensifying stress, weakening social support, and reducing access to protective environments, thereby amplifying both environmental and biological vulnerabilities.

To explore potential heterogeneity in effect estimates by age, we conducted stratified analyses comparing patients younger than 65 years with those aged 65 years and older. Overall, we did not observe clear evidence of effect modification by age, as effect estimates were broadly similar across groups and confidence intervals largely overlapped. Among individuals aged 65 years and older, the associations between CSI and hospital visits for anxiety, mood, and adjustment disorders showed some deviations from linearity. Associations for anxiety and adjustment disorders were stronger at higher CSI values among those aged 65 years and older, although estimates were less precise. Given the large number of models examined, these differences should be interpreted cautiously. Overall, these findings suggest that age did not substantially modify the associations between community severance and mental health-related hospital visits in our sample.

Our findings, nonetheless, should be interpreted in light of our limitations. The use of data on hospital visits means that we likely captured only the most severe mental health cases, missing subclinical or less severe cases. Consequently, our findings may underestimate the true burden of community severance on the full spectrum of mental health. In addition, we only had access to aggregated data on hospital visits without unique patient identifiers, which prevented us from distinguishing repeat hospital visits by the same individual from visits by different individuals. Consequently, recurrent admissions may contribute to overdispersion in the outcome counts. While our quasi-Poisson models allow the variance to exceed the mean and help account for extra variability when estimating standard errors, they do not allow us to identify the source of this overdispersion. In the absence of information on repeat hospital visits, we cannot determine whether the probability of recurrent admissions varies systematically with CSI after accounting for the covariates included in our models (e.g., SES, density). Therefore, the potential impact of this limitation on the direction of the associations remains unclear. Also, residential location was based on the address recorded at the time of hospital visit, and we lacked information on residential history or length of residence, potentially introducing exposure measurement error. Because individuals with mental health disorders may experience substantially higher residential mobility than the general population,^69–71^ this error could be differential. However, without information on whether movers typically transition to higher or lower CSI environments, and noting that mobility in this population is often characterized by “downward drift” into disadvantaged areas or “involuntary churning” through institutional settings,^69,72^ the direction and magnitude of this potential bias remain uncertain. Also, while we considered air pollution as one potential pathway for the CSI–mental health association, other correlated exposures, such as traffic noise, may also mediate this association.

As vehicle fleet electrification progresses and traffic emissions continue to decline, it is critical for future investigations to examine how the presence of road infrastructure and motorized vehicles influences mental well-being, particularly in a warming climate where synergistic stressors like extreme heat also contribute to mental health risks.^73^ Future research should also leverage cohort data to explore symptomatology and incidence of mental health disorders. Moreover, studies should also explore in situ experiences through walking interviews or Global Positioning System-based qualitative assessments (e.g., photovoice)^74^ to better understand how people experience communities severed by the presence of roads and motorized vehicles, and how such experiences may influence mental health.

## Conclusions

Our study indicates community severance related to roads and motorized traffic as an urban characteristic that may be relevant for understanding variation in schizophrenia-related hospital visits. We found that areas with greater community severance were associated with higher rates of schizophrenia-related hospital visits. The association for schizophrenia persisted even after accounting for potential confounders and blocking the potentially mediating pathway through traffic-related air pollution, suggesting a distinct impact of the presence of road infrastructure and motorized vehicles in cities on mental health. By addressing structural barriers that divide communities, cities may reduce these risks and promote mental well-being in urban populations.

## Conflicts of interest

The authors declare that they have no conflicts of interest with regard to the content of this report.

## Supplementary Material


