# Supplementary material for: Community severance and mental health-related hospital visits in New York City
Source: Environ Epidemiol. 2026 Apr 27;10(3):e482. doi: 10.1097/EE9.0000000000000482 (PMC13124370; doi:10.1097/EE9.0000000000000482)
Supplement: Supplementary file 1 [file ee9-10-e482-s001.pdf]

Supplemental Material

Community severance and mental health-related hospital visits in New York City

Jaime Benavides, Gali Cohen, Jeff Goldsmith, and Marianthi-Anna Kioumourtzoglou

Table of Contents

Text S1. Description of data inputs for Community Severance Index 2011-2014 estimation.
Table S1. Causes of hospital visits used in the analysis with ICD-9-CM codes.
Table S2. Estimated linear associations in the main analysis.
Table S3. Estimated linear associations in the sensitivity analysis, adding outliers.
Table S4. Estimated linear associations in the secondary analysis, adding black carbon.
Figure S1. Directed Acyclic Graph (DAG).
Figure S2. Factor loadings for the main pattern of road and pedestrian infrastructure, and road traffic activity.
Figure S3. Spearman correlation coefficients among covariates and exposure.
Figure S4. Maps of covariates.
Figure S5. Linear associations between Community Severance Index in ZIP code and mental health hospital visits when adding Community Severance Index outliers.
Figure S6. Non-linear association between Community Severance Index in ZIP code and mood disorder mental health hospital visits when adding Community Severance Index outliers.
Figure S7. Associations between ZIP code-level Community Severance Index (CSI) and mental health hospital visits when adding black carbon concentrations.
Figure S8. Associations between ZIP code-level community severance index (CSI) and cause-specific mental health hospital visits when stratifying by age.

Text S1. Description of data inputs for the estimation of the 2011-2014 community severance index.

Using the same conceptual and analytical framework, we re-estimated the CSI for the 2011–2014 period. To maximize consistency with the CSI construction for 2019 (Benavides et al., 2024), we relied on the same data categories and, whenever possible, the same data sources, selecting versions representative of the earlier timeframe. These included the Smart Location Database (SLD) version 2, published in 2014, which captures built environment and transportation characteristics for the 2011–2014 period (US-EPA, 2014). Road infrastructure, traffic volume, proximity, and barrier factor variables were derived from Highway Performance Monitoring System (HPMS) data for New York State from 2012 (US-DOT, 2012) which serves as the predecessor to the Freight Analysis Framework Version 5 (FAF5) dataset used for the 2019 CSI and provides comparable roadway functional classification and traffic information (US-DOT, 2023). Traffic-related emissions were represented using DARTE CO2 emissions data for 2012 (Gately et al., 2019) to ensure temporal alignment across inputs. Using these harmonized datasets, we re-ran the Principal Component Pursuit (PCP) and, subsequently, factor analysis. For further details on the construction of the CSI, we refer to Benavides et al. (2024).

References:

US-EPA. Smart location database. version 2.0 user guide. 2014. https://www.epa.gov/sites/default/files/2014-03/documents/sld_userguide.pdf

USDOT. Highway performance monitoring system (HPMS) - 2012 New York, 2012. https://catalog.data.gov/dataset/highway-performance-monitoring-system-hpms-2012-new-york.

US-DOT BTS. Freight analysis framework, 2023. URL https://www.bts.gov/faf.

C Gately, LR Hutyra, and IS Wing. DARTE annual on-road CO2. ORNL DAAC, Oak Ridge, Tennessee, USA, 2019. https://daac.ornl.gov/CMS/guides/CMS_DARTE_V2.html.

J. Benavides, S. Usmani, V. Kumar, and M.-A. Kioumourtzoglou. Development of a community severance index for urban areas in the United States : A case study in New York City. Environment International, 185:108526, 2024. ISSN 0160-4120. doi: 10.1016/j.envint.2024.108526.

Table S1. Causes of hospital visits used in the analysis with ICD-9-CM codes.

Cause of hospital visit	ICD-9-CM
Mood disorder	293.83, 296.00, 296.01, 296.02, 296.03, 296.04, 296.05, 296.06, 296.10, 296.11, 296.12, 296.13, 296.14, 296.15, 296.16, 296.20, 296.21, 296.22, 296.23, 296.24, 296.25, 296.26, 296.30, 296.31, 296.32, 296.33, 296.34, 296.35, 296.36, 296.40, 296.41, 296.42, 296.43, 296.44, 296.45, 296.46, 296.50, 296.51, 296.52, 296.53, 296.54, 296.55, 296.56, 296.60, 296.61, 296.62, 296.63, 296.64, 296.65, 296.66, 296.7, 296.80, 296.81, 296.82, 296.89, 296.90, 296.99, 300.4, 311.0
Anxiety disorder	293.84, 300.00, 300.01, 300.02, 300.09, 300.10, 300.20, 300.21, 300.22, 300.23, 300.29, 300.3, 300.5, 300.89, 300.9, 308.0, 308.1, 308.2, 308.3, 308.4, 308.9, 309.81, 313.0, 313.1, 313.21, 313.22, 313.3, 313.82, 313.83
Adjustment disorder	309.0, 309.1, 309.22, 309.23, 309.24, 309.28, 309.29, 309.3, 309.4, 309.82, 309.83, 309.89, 309.9
Schizophrenia and other psychotic disorders	293.81, 293.82, 295.00, 295.01, 295.02, 295.03, 295.04, 295.05, 295.10, 295.11, 295.12, 295.13, 295.14, 295.15, 295.20, 295.21, 295.22, 295.23, 295.24, 295.25, 295.30, 295.31, 295.32, 295.33, 295.34, 295.35, 295.40, 295.41, 295.42, 295.43, 295.44, 295.45, 295.50, 295.51, 295.52, 295.53, 295.54, 295.55, 295.60, 295.61, 295.62, 295.63, 295.64, 295.65, 295.70, 295.71, 295.72, 295.73, 295.74, 295.75, 295.80, 295.81, 295.82, 295.83, 295.84, 295.85, 295.90, 295.91, 295.92, 295.93, 295.94, 295.95, 297.0, 297.1, 297.2, 297.3, 297.8, 297.9, 298.0, 298.1, 298.2, 298.3, 298.4, 298.8, 298.9

Table S2. Estimated linear associations for community severance and each mental health disorder, presented as rate ratios (RR) per interquartile range (IQR) increase in community severance index (CSI), adjusting for covariates in the main analysis.

Mental health disorder	Rate ratio	Confidence intervals (95%)
Mood	1.07	(0.98, 1.16)
Anxiety	1.06	(0.99, 1.14)
Adjustment	1.07	(0.97, 1.19)
Schizophrenia	1.13	(1.00, 1.27)

Table S3. Estimated linear associations between community severance and each mental health disorder, presented as rate ratios (RR) per interquartile range (IQR) increase in community severance index (CSI), adjusting for covariates, in the sensitivity analysis including potential outliers. The association with mood disorders in this sensitivity analysis deviated from linearity and is presented in Figure S6.

Mental health disorder	Rate ratio	Confidence intervals (95%)
Anxiety	1.08	(1.02, 1.15)
Adjustment	1.09	(0.99, 1.21)
Schizophrenia	1.13	(1.00, 1.13)

Table S4. Estimated linear associations for each mental health disorder and community severance, adjusting for covariates in the secondary analysis when including black carbon and year.

Mental health disorder	Rate ratio	Confidence intervals (95%)
Mood	1.06	(0.97, 1.15)
Anxiety	1.04	(0.96, 1.11)
Adjustment	1.07	(0.96, 1.19)
Schizophrenia	1.11	(0.98, 1.26)

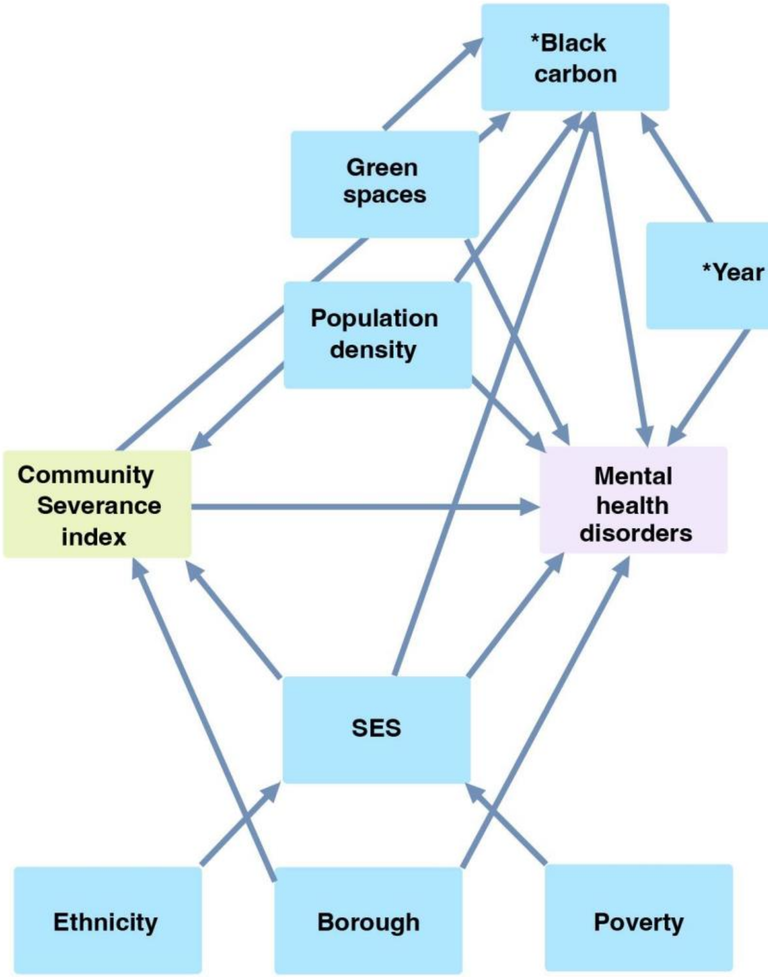

Figure S1. Directed Acyclic Graph (DAG) explaining the relationship between Community Severance Index (CSI) and mental health disorders in New York City. This model was used in the present study to evaluate the effect of CSI on mental health disorders (green and pink boxes). Covariates and potential confounders are shown in blue boxes. In this study, neighborhood Socioeconomic Status (SES) is a latent construct represented by its descendants' ethnicity and poverty, which we used as proxies of neighborhood SES in analyses. *Black carbon is treated as a potential mediator in this study, and it is included in the secondary analysis, along with calendar year.

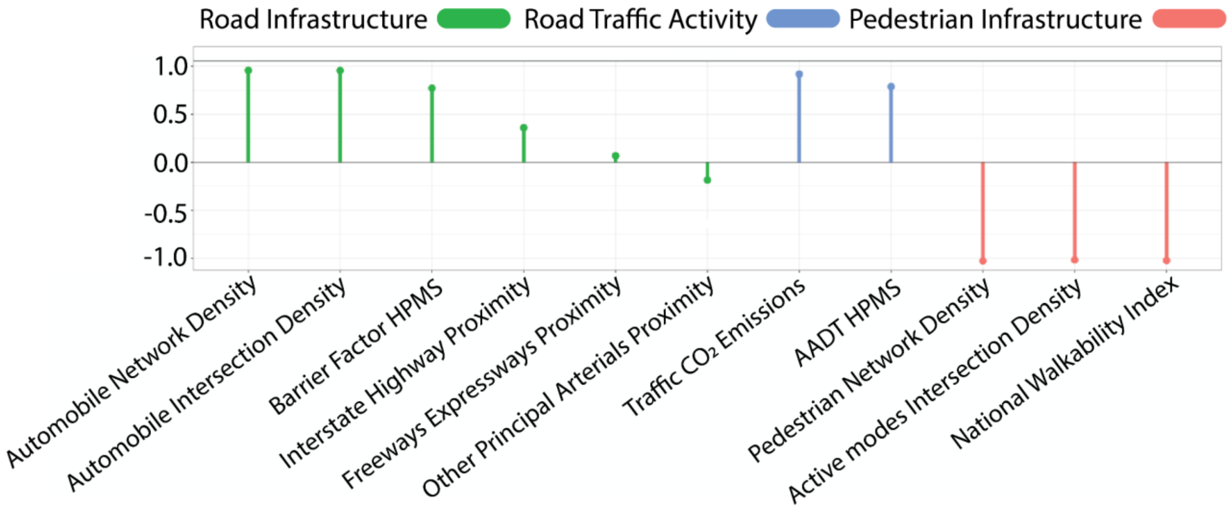

Figure S2. PCP-FA Factor 1 loadings. This factor explained 75% of the shared variability in the PCP low-rank matrix. We interpreted it as the community severance index, as road traffic activity and road infrastructure positively and strongly load on this factor, while pedestrian infrastructure negatively loads on it. The complete names for the variables are (in order, from left to right): Automobile network density; Automobile intersection density; Barrier factor; Interstate highway proximity; Freeways / expressways proximity; Principal arterial – other proximity; Traffic CO2 emissions; Annual Average Daily Traffic (AADT), Pedestrian network density; Street intersection density (no automobile); and National Walkability Index.

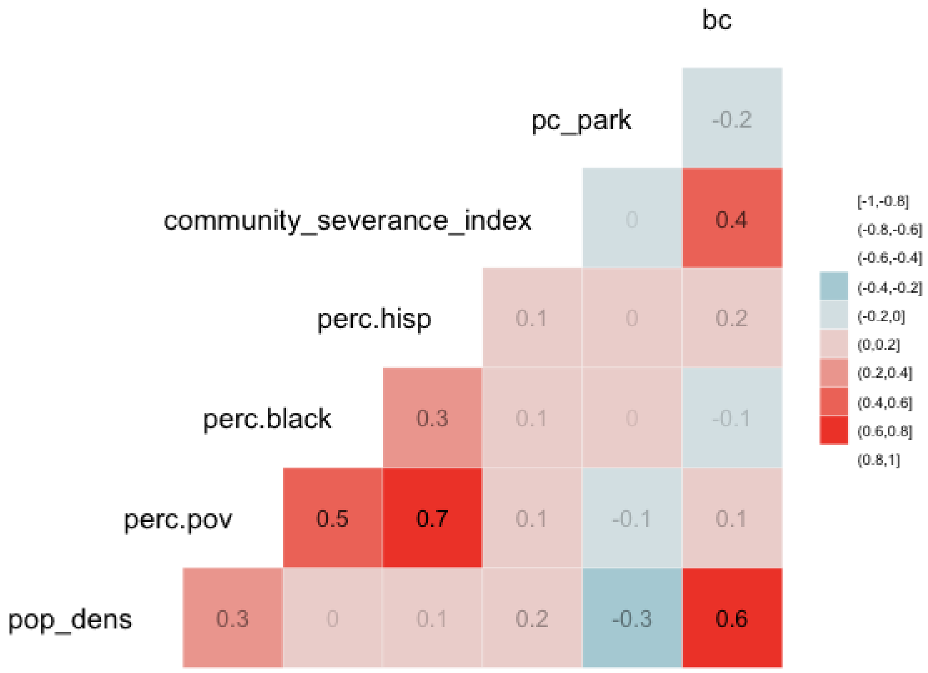

Figure S3. Spearman correlation coefficients among Community Severance Index and covariates. bc: Black Carbon; pc_park.: Green Space access (%); perc_hisp: Hispanic (%); perc_black: Black (%); perc_pov: Poverty rate (%); pop_dens: Population density.

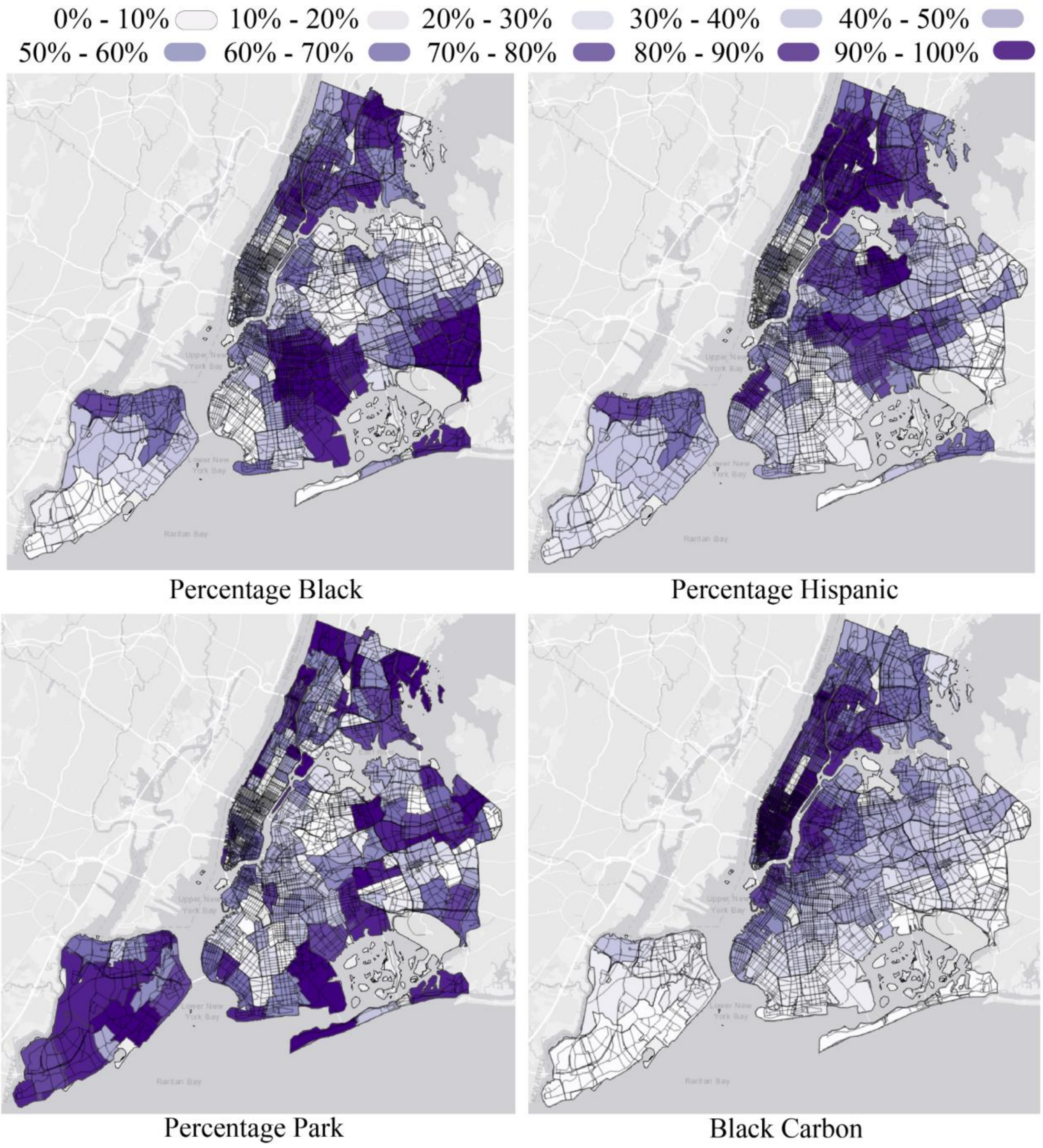

Figure S4. Maps of the spatial distribution of ZIP code-level covariates for percentage Black, percentage Hispanic (top panel), along with percentage park (green spaces), and black carbon (bottom panel). Values of these variables are presented in deciles.

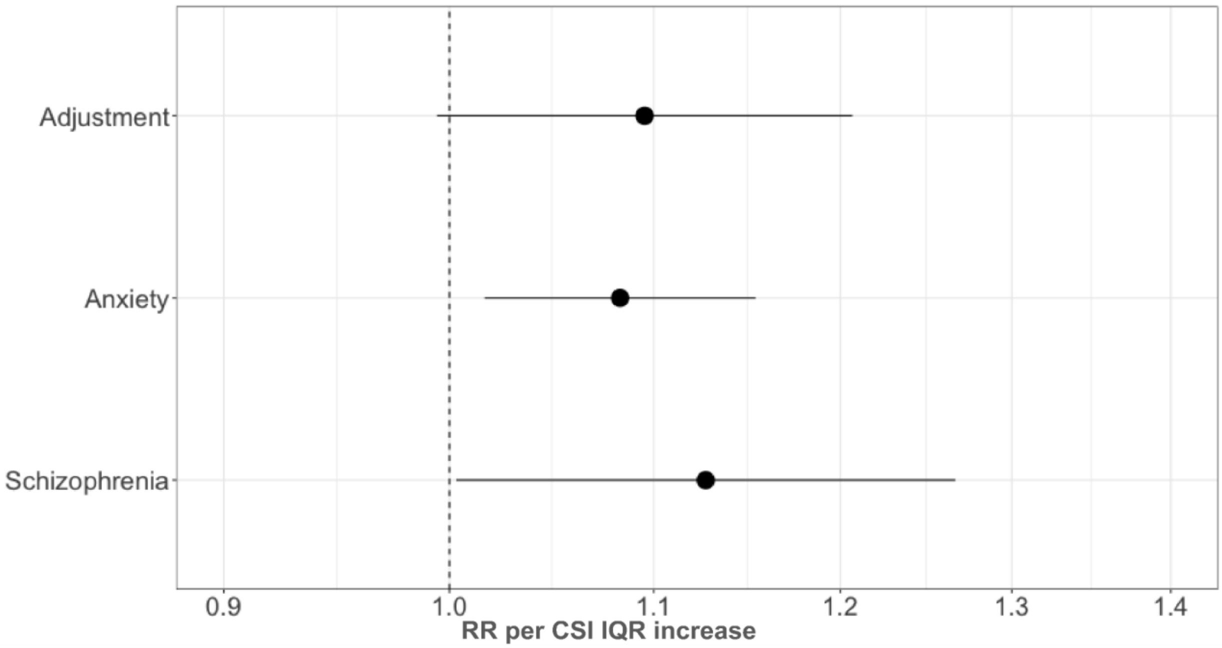

Figure S5. Associations between ZIP code-level community severance index (CSI) and mental health hospital visits for adjustment, anxiety, and schizophrenia disorders when including CSI outliers in the sensitivity analysis. The vertical dotted line represents the null; horizontal lines are the 95% confidence intervals for each estimated rate ratio (RR). The x-axis shows RRs per interquartile range (IQR) increase in CSI and it is plotted on a logarithmic scale. Models were adjusted for population density, Black (%), Hispanic (%), poverty rate, borough, and green spaces. See Supplemental Table S3 for corresponding numeric data. The association with mood disorders in this sensitivity analysis deviated from linearity and is presented in Figure S6.

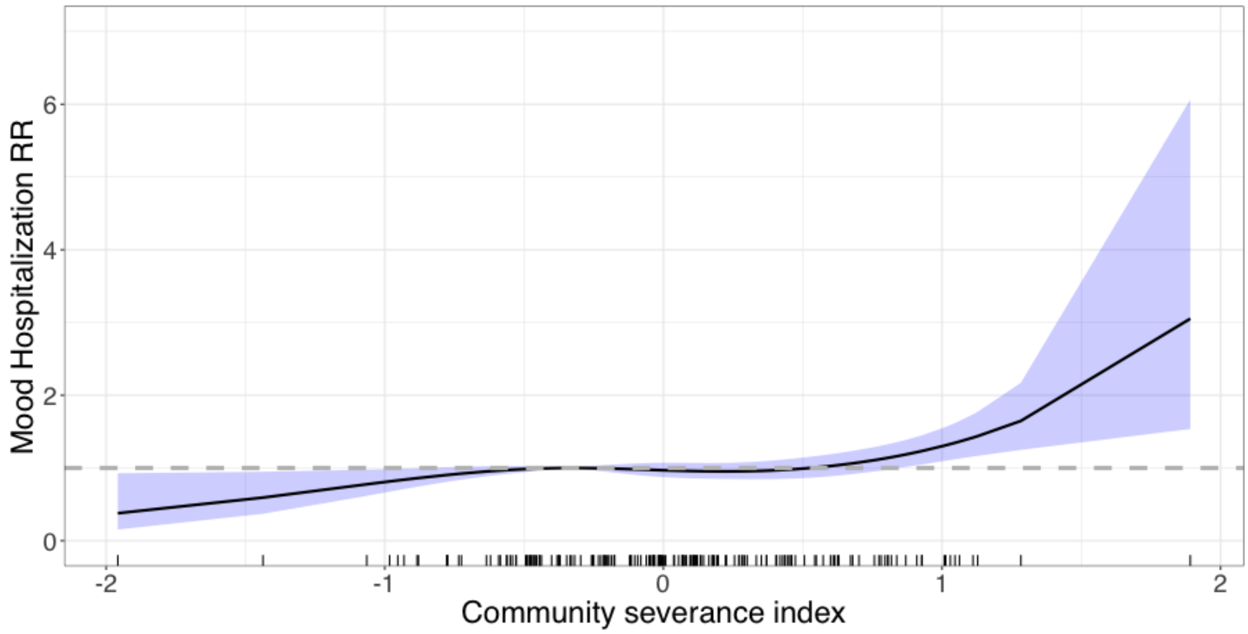

Figure S6. Associations between ZIP code-level community severance index (CSI) and mental health hospital visits for mood disorder when including CSI outliers to the main analysis, modeled using a natural spline with 4 degrees of freedom. The 25th percentile of CSI was set as the reference value for plotting the results. The horizontal dotted line represents the null (RR=1).

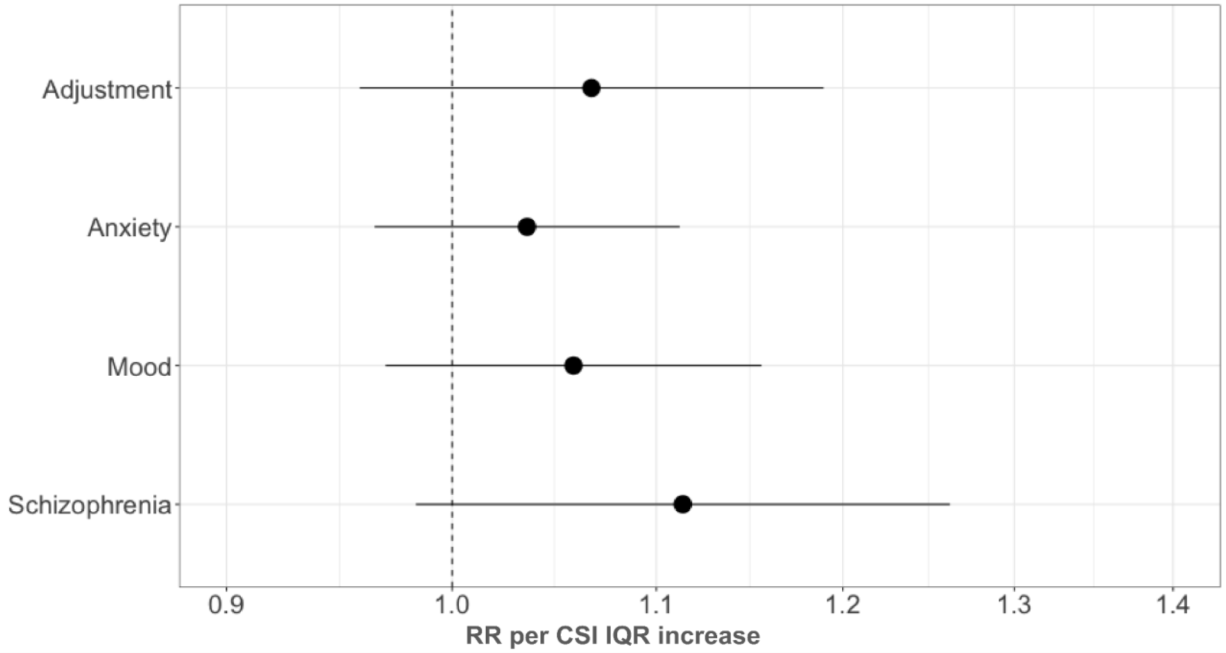

Figure S7. Associations between ZIP code-level community severance index (CSI) and mental health hospital visits when adding black carbon concentrations as a covariate to the main analysis. The vertical dotted line represents the null. Models were adjusted for population density, race/ethnicity, poverty rate, borough, CSI, and green spaces. See Supplemental Table S4 for corresponding numeric data.

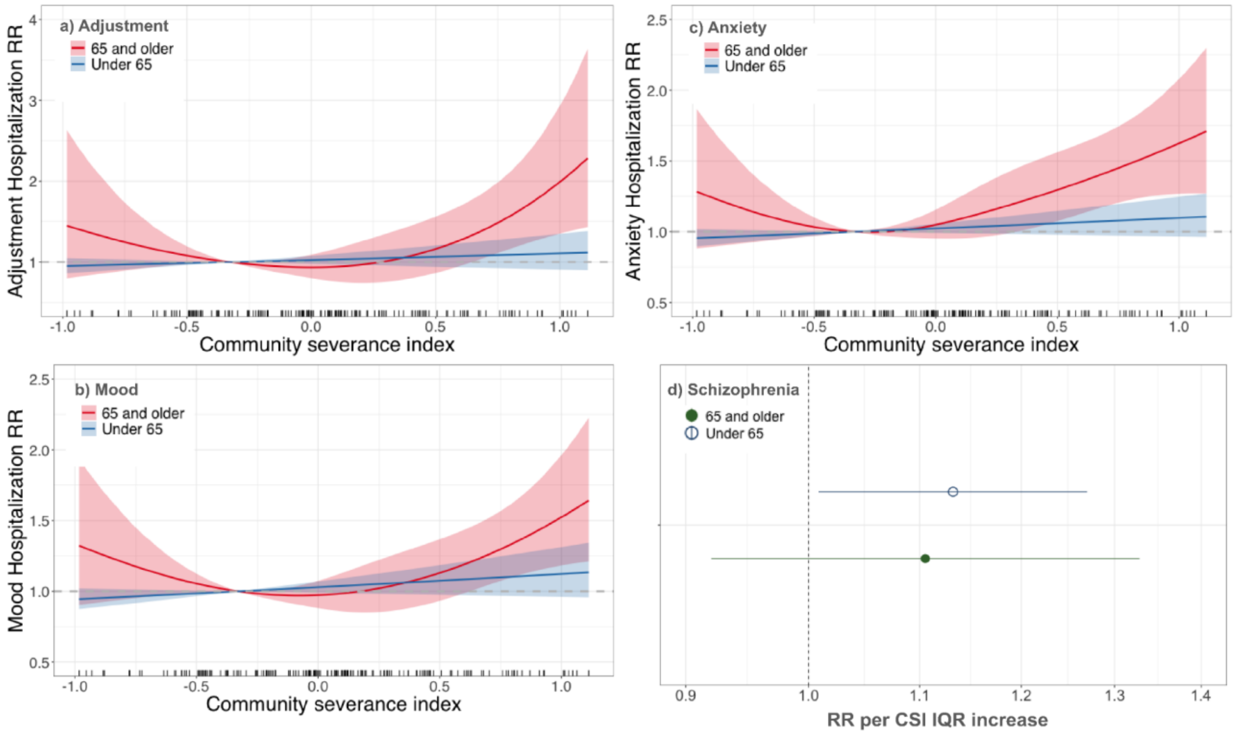

Figure S8. Associations between ZIP code-level community severance index (CSI) and cause-specific mental health hospital visits when stratifying by age (under 65 vs 65 and older). For adjustment-, mood-, and anxiety-related visits, we detected deviations from linearity in the aged 65 and older stratum. In the panels presenting the results for those outcomes, the 25th percentile of the CSI was set as the reference. The horizontal dotted line represents the null (rate ratio, RR=1). The estimated associations for schizophrenia were linear in both strata. In panel (d), showing the schizophrenia estimates, the vertical dotted line represents the null; horizontal lines are the 95% confidence intervals for the estimated, stratum-specific RRs, shown for each interquartile range (IQR) increase in CSI, plotted on a logarithmic scale. Models were adjusted for population density, Black (%), Hispanic (%), poverty rate, borough, and green spaces similar to the main analysis.
